# Microgel assisted Lab-on-Fiber Optrode

**DOI:** 10.1038/s41598-017-14852-5

**Published:** 2017-10-31

**Authors:** A. Aliberti, A. Ricciardi, M. Giaquinto, A. Micco, E. Bobeico, V. La Ferrara, M. Ruvo, A. Cutolo, A. Cusano

**Affiliations:** 1 Optoelectronics Group, Department of Engineering, University of Sannio, I-82100 Benevento, Italy; 2 ENEA, Portici Research Center, P.le E. Fermi 1, I-80055 Portici, Napoli, Italy; 3 Institute of Biostructure and Bioimaging, National Research Council, I-80143 Napoli, Italy

## Abstract

Precision medicine is continuously demanding for novel point of care systems, potentially exploitable also for *in-vivo* analysis. Biosensing probes based on Lab-On-Fiber Technology have been recently developed to meet these challenges. However, devices exploiting standard label-free approaches (based on ligand/target molecule interaction) suffer from low sensitivity in all cases where the detection of small molecules at low concentrations is needed. Here we report on a platform developed through the combination of Lab-On-Fiber probes with microgels, which are directly integrated onto the resonant plasmonic nanostructure realized on the fiber tip. In response to binding events, the microgel network concentrates the target molecule and amplifies the optical response, leading to remarkable sensitivity enhancement. Moreover, by acting on the microgel degrees of freedom such as concentration and operating temperature, it is possible to control the limit of detection, tune the working range as well as the response time of the probe. These unique characteristics pave the way for advanced label-free biosensing platforms, suitably reconfigurable depending on the specific application.

## Introduction

In biochemical sensing field, Lab-on -Fiber (LOF) based devices essentially consist on the combination of optical resonant nanostructures (typically patterned metallic slab supporting surface plasmon resonances (SPR)) and functional coating materials integrated on the optical fiber tip^1–7^. LOF technology is continuously leading to the development of novel biosensing probes with unique properties in term of size, weight and ease of interrogation^4,5^. In addition to point of care applications, LOF based devices seem to be particularly promising for *in-vivo* analysis systems, thanks to the intrinsic properties of optical fibers that make them easily integrable inside medical catheters or needles^8^. Typically, the working principle of LOF probes relies on the affinity interaction of a ligand attached to the sensor surface with the target molecule present in a liquid solution at a certain concentration. However, standard label-free approaches fail when target molecules are small, for example about a few hundreds of dalton. In that case, the ligand/analyte binding process produces a biological layer that is not thick enough for providing a local refractive index (RI) change that is optically detectable by the sensor. Analogous issues occur in such applications where the detection of larger analytes with very low limit of detection (LOD) is required. To enhance the sensitivity, gold and magnetic nanoparticles have been proposed as “molecular concentrators” able to localize multiple binding events on a single particle, and successively deliver target analyte from the solution to the sensor surface^9–11^. At the same time, approaches exploiting hydrogels (HGs) have been proposed^12,13^. HGs basically allow to: i) increase the analyte loading capacity by translating a conventional 2D interaction surface into a 3D volume interaction (thus favoring *concentration* effect), ii) amplify the signal transduction by changing the degree of HGs swelling due to detection/recognition events. HGs have been integrated both around^14–16^ and on top of optical fiber^17–19^. However, conventional HGs have limited utility in practical applications due to the fact that the rate of stimuli response is inversely proportional to the square of the size of the gel^20^. For this reason, in the last years, scientific community has focused the attention on *microgels* (MGs) i.e. colloidal HGs microsized particles with radius in the range between tens of nanometers and few microns^21–27^. The use of MGs is also advantageous when morphological or spatial control over the film is required, or when bulk polymerization approaches of HGs fail^28^. By exploiting combination of MGs with photonic chips, detection of pH^29^, glucose^30^, protein^31^ and specific DNA sequences^32^ have been reported.

**Figure 1:**
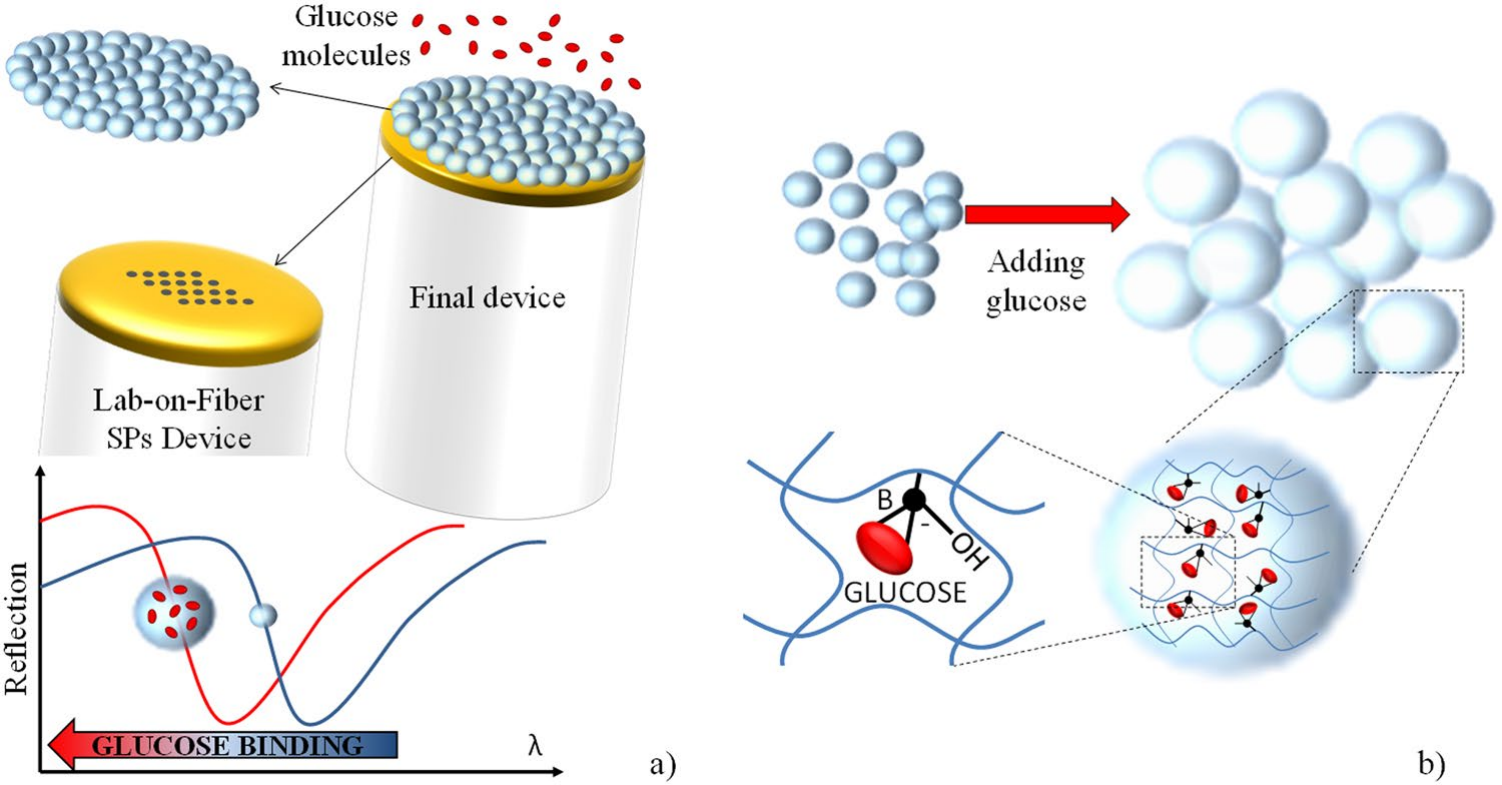
Schematic of the developed device based on the combination of MGs and LOF Technology. In the presence of a molecular binding event the SPR dip in the reflection spectrum blue-shifts due to the changes in the polymeric network density of the MGs (**a**). Schematic view of the glucose binding event inside the MGs network (**b**).

In a pioneer study, we have proposed a biosensing platform consisting of a MGs layer baked by a metal nanostructure integrated on the optical fiber facet (see Fig. 1a)^33,34^. As case of study, for estimating the optical response amplification of the proposed device, a well-know benchmark for the class of small-molecules such as glucose has been used. In the wake of this proof of principle demonstration, here we exploit all the degrees of freedom offered by the combination of MGs with LOF optrode to tailor the sensing performances in terms of LOD, working range as well as response time. Specifically, we demonstrate that probes obtained with different MGs concentration exhibit different behaviors when interacting with target molecules. In particular, we find that, by selecting a suitable MGs concentration, it is possible to tune the probe sensitivity range and thus the LOD. We also study the impact of MGs parameters (in particular of the temperature) on the detection time response, which represents another important parameter of merit for biosensing applications. Competition and/or selectivity tests go beyond the scope of this work. Overall our results demonstrate that by combining LOF devices and MGs it is possible to develop advanced and flexible label-free fiber optic nanoprobes with exciting performances, particularly useful in the cases of small-molecule detection.

## Methods

### Materials

N-isopropylacrylamide, N,N′-methylenebisacrylamide (BIS, 99%), acrylic acid (AAc, 99%), ammonium persulfate (APS, 98%), α-D-glucose, and 3-amino phenyl boronic acid (APBA) hydrochloride (98%), Sodium dodecyl sulfate (SDS), 11-mercaptoundecanoic acid, sodium bicarbonate (NaHCO3) and sodium carbonate (Na2CO3) were purchased from Sigma Aldrich and used without further purification. 1-Ethyl-3-(3-dimethylaminopropyl) carbodiimide hydrochoride (EDC) and BupH 2-(Nmorpholino) ethanesulfonic acid (MES) buffered saline were purchased from Pierce through Thermo Scientific and used without further purification. All deionized water was obtained from a Milli-Q Plus system from Millipore.

### LOF probe fabrication

A 35 nm thick Au film has been deposited by means of electron beam evaporation on the cleaved end of a standard single mode optical fiber (Corning SMF-28). A thin (~2 nm) chromium film has been used for improving adhesion. The square lattice of holes has been patterned on gold film by using the FEI Quanta 200 3D system. Beam currents and accelerating voltages have been set to 30 pA and 30 kV respectively.

### MGs synthesis and functionalization

A 3-neck flask was equipped with a reflux condenser, nitrogen inlet, and temperature probe, and filled with a solution of NIPAm (11.1 mmol), BIS (0.652 mmol) and SDS (0.035 g) in 99 mL deionized water. The monomers solution was heated to 70 °C over ~1 hour and purged with N_2_. AAc (1,3 mmol) was then introduced in to heated reaction solution with a single injection step. A solution of APS (0,3 mmol) in 1 mL of deionized water was added to initiate the polymerization and the reaction was allowed to proceed at 70 °C for 4 hours under nitrogen. The MGs suspension was cooled overnight and then collected into rehydrated dialysis tubing (12–14k nominal MWCO) for purification. The tubes were immersed into a deionized water solution and for 15 days the water was changed 2x daily. The cleaned P(NIPAM-AAc) MGs were recombined and lyofilized.

MGs were functionalized with boronic moieties using APBA under EDC activation in pH 4,7 MES buffer according to the procedure described in ref.^35^ (see Supplementary Information, section S1).

### Integration of MGs Film on the LOF probe

The fiber tip was immersed into 200 µL aliquot of MGs solution at concentration of 5%, 0.5%, 0.05% for 1 h, the film was then allowed to dry at 37 °C into an oven for 1 hour and soaked overnight at 30 °C in a deionized water bath. Finally, the deposited MGs film was dried in air. By controlling dipping and extraction speed, temperature solution, drying environment conditions (humidity and temperature), it is possible to achieve a good repeatability of the fabricated probes.

Concerning functionalization, the probes were placed in pH 4.7 MES buffered for 1 h at 4 °C into the refrigerator and a solution of 250 mM EDC and 125 mM APBA was added to the buffer. The reaction was allowed to proceed overnight at 4 °C. All samples were rinsed and soaked in deionized water for 2 days to remove any unreacted reagents.

## Results and Discussion

### LOF probe design and fabrication

The LOF probe consists of a gold layer integrated on the fiber tip and structured with a square lattice of holes in such a way to enhance extraordinary optical transmission (EOT) through the excitation of SPR, corresponding to a dip in the reflection spectrum^36–38^. The probe design is based on full wave simulations carried out by means of finite element method software (Comsol Multiphysics – RF module). Details on the numerical simulations can be found in the Supplementary Information (section S2). The lattice period *a*, the holes radius *r* and the gold layer thickness are 650 nm, 195 nm and 35 nm respectively. These parameters have been chosen in order (i) to set the resonance wavelength in the operating range of standard single mode fibers and (ii) to achieve a good compromise between resonance bandwidth and surface sensitivity. The device has been fabricated by means of a focused ion beam (FIB) milling process directly applied to the gold layer deposited on the fiber tip. Figure S3.2b shows the SEM image (top view) of the fabricated device. The experimental reflectance spectrum is shown in Fig. S3.2b (dashed red line) and it is in good agreement with the numerical prediction. A schematic and a description of the interrogation setup can be found in the Supplementary Information (section S3).

### Microgels synthesis, functionalization and characterization

(pNIPAm-co-AAc) MGs were prepared by following a procedure defined in ref.^39^. Successively, for making MGs sensitive to glucose, they were functionalized with boronic moieties using APBA under EDC catalysis in pH 4,7 MES buffer^35^. Negative control samples were similarly prepared by using (pNIPAM-co-AAc) MGs treated with APBA only (without EDC) in pH 4.7 buffer. MGs were morphologically characterized by means of dynamic light scattering (DLS) measurements which allow to determine their hydrodynamic radius (R_h_) in buffer solution (see Supplementary Information, section S4).

**Figure 2:**
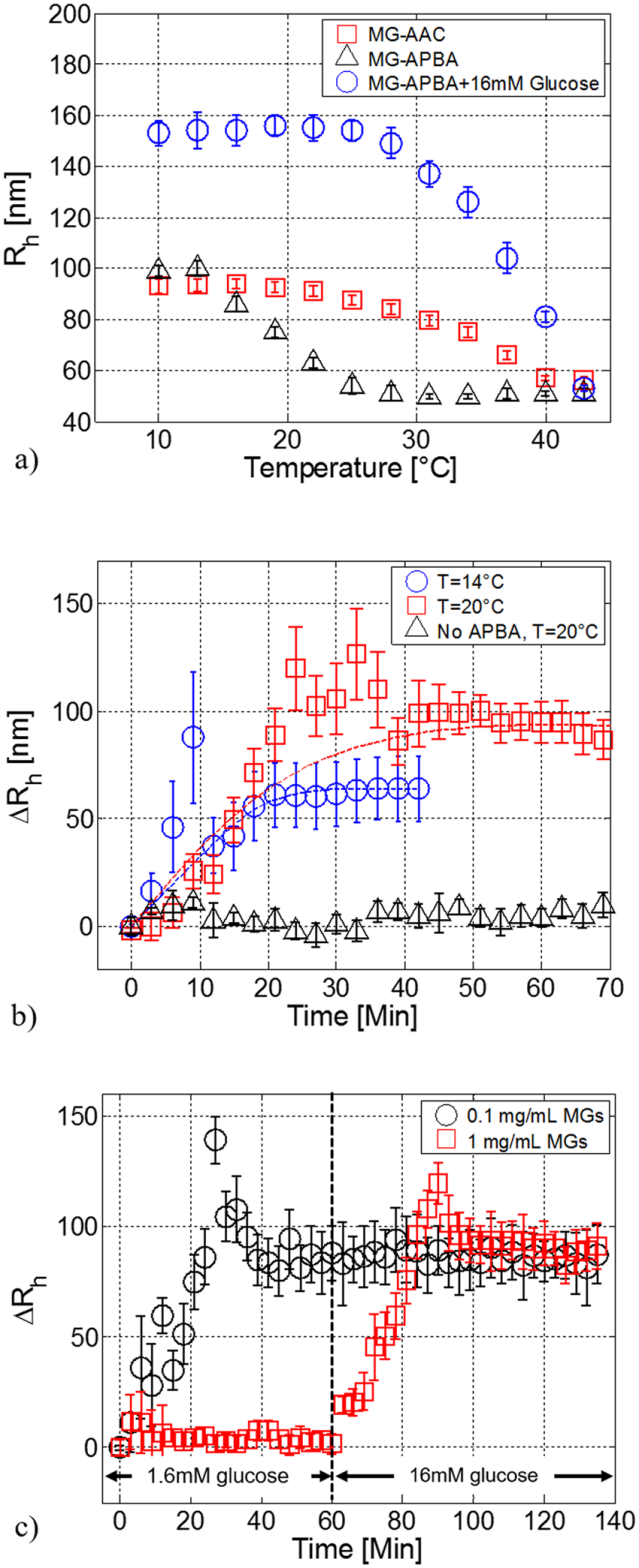
DLS measurements of functionalized MGs. Hydrodynamic radius (R_h_) of the native (pNIPAm-co-AAc) MGs (red squares) and functionalized (pNIPAm-APBA) MGs before (black triangles) and after glucose binding (blue circles) as a function of temperature (**a**), R_h_ variations (R_h_ - R_0_) of the (pNIPAm-APBA) MGs as function of time during glucose binding at 16 mM, for temperatures of 14 (blue) and 20 °C (red), black triangles curve represents R_h_ variations for not functionalized MGs (negative control) (**b**). R_h_ variations as a function of time for MGs concentration of 1 mg/mL and 0.1 mg/mL for glucose concentrations of 16 mM and 1.6 mM at 20 °C (**c**).

Figure 2a shows the variations of R_h_ induced by temperature, before (red squares), after (black triangles) functionalization with APBA, and after glucose binding at 20 °C. The experimental uncertainties represent the standard error of the mean of 5 replicate runs. As shown in Fig. 2a (red squares curve), native (pNIPAM-co-AAc) MGs undergo sharp volume phase transition at ~32 °C, corresponding to the volume phase transition temperature (VPTT); at lower temperatures, (pNIPAM-co-AAc) MGs have R_h_∼90 nm, that reduces down to ∼50 nm at high temperatures.

After modification with APBA (black triangles curve), (pNIPAM-APBA) MGs exhibit a VPTT of 17 °C, which is about 15 °C lower than that of the native (pNIPAM-co-AAc) MGs. The VPTT shift is due to the increase of the hydrophobic functionalities caused by the conjugation of the APBA to the native MGs^35^. Upon exposure to glucose solution, the (pNIPAM-APBA + 16 mM Glucose) curve (blue circles) moves towards higher temperature, exhibiting a VPTT of 35 °C. This shift is due to the formation of a glucose-boronate ester that makes the polymer more hydrophilic, thus influencing the glucose swelling response.

For evaluating the influence of MGs temperature, glucose binding experiments have been repeated at both 14 °C and 20 °C, corresponding to different MGs states, i.e. swollen and partially collapsed respectively. The results shown in Fig. 2b reveal a trade-off between achievable MGs size variation and response time. As mentioned above, glucose binding is expected to induce a MGs swelling (Fig. 1b). For maximizing the R_h_ variations, the buffer solution temperature should be kept at those values for which MGs are in the fully collapsed state. However, in the high temperature range, the hydrophobic interactions driving thermal gel collapse tend to prevail on the ionization driving force of glucose-induced swelling^30–35^. In fact, when MGs are not completely swollen (i.e. at 20 °C), R_h_ variations are maximum (ΔR_h_ = ~90 nm); however, the partially collapsed state of the MGs makes the glucose molecules diffusion inside the polymeric network difficult, thus slowing down the response time. Clearly, an opposite situation occurs in the small temperature regime (T = 14 °C) where ΔR_h_ and response time are found to be 65 nm and ~30 min respectively. The ΔR_h_ values for the two temperatures are in line with the differences between (pNIPAM-APBA) MGs volume phase transition curves before and after exposure to 16 mM glucose concentration (Fig. 2a).

We have also analyzed, by means of DLS measurements, the relationship between the MGs concentration in solution and the detectable glucose concentrations. Results shown in Fig. 2c show that MGs at concentration of 0.1 mg/mL respond to a glucose concentration of 0.16 mM, and then they reach the saturation level. On the other hand, MGs at 1 mg/ml are not able to sense the same glucose concentration (0.16 mM), but respond to that of one order of magnitude higher (1.6 mM). This could be explained by considering that, as the MG concentration decreases, also do the number of ligand molecules, consequently, a lower glucose concentration is sufficient to saturate all the binding sites of the MGs network. The same hydrodynamic radius change measured for the two different MGs concentrations is due to the fact that in both the cases the MGs swelling has reached the saturation level. Since MGs particles dissolved in solution are fully swollen at those glucose concentrations, the DLS measurement of the particles average radius size of the two solutions gives the same result. We have also repeated the binding experiments with not functionalized MGs (i.e. treated only with APBA without EDC). As expected, no MGs size variations after glucose addition have been observed.

### Integration of MGs Film on the LOF probe

MGs films have been painted onto the patterned Au layer integrated on the fiber tip by means of a dip coating technique (see Methods section). After depositing MGs, they were functionalized with APBA^30^. Control samples were prepared with a similar procedure by using (pNIPAm-co-AAc) MGs films treated only with APBA (without EDC).

In order to study the impact of the MG film properties on the glucose detection amplification performances, we have fabricated and tested three probes constituted by the *same* LOF device, but coated by MGs layers corresponding to three concentrations (5, 0.5 and 0.05%) of MGs in water solution. Obviously, different concentrations of MGs solutions give rise to thin films characterized by different degrees of homogeneity, uniformity, density, and compactness.

**Figure 3:**
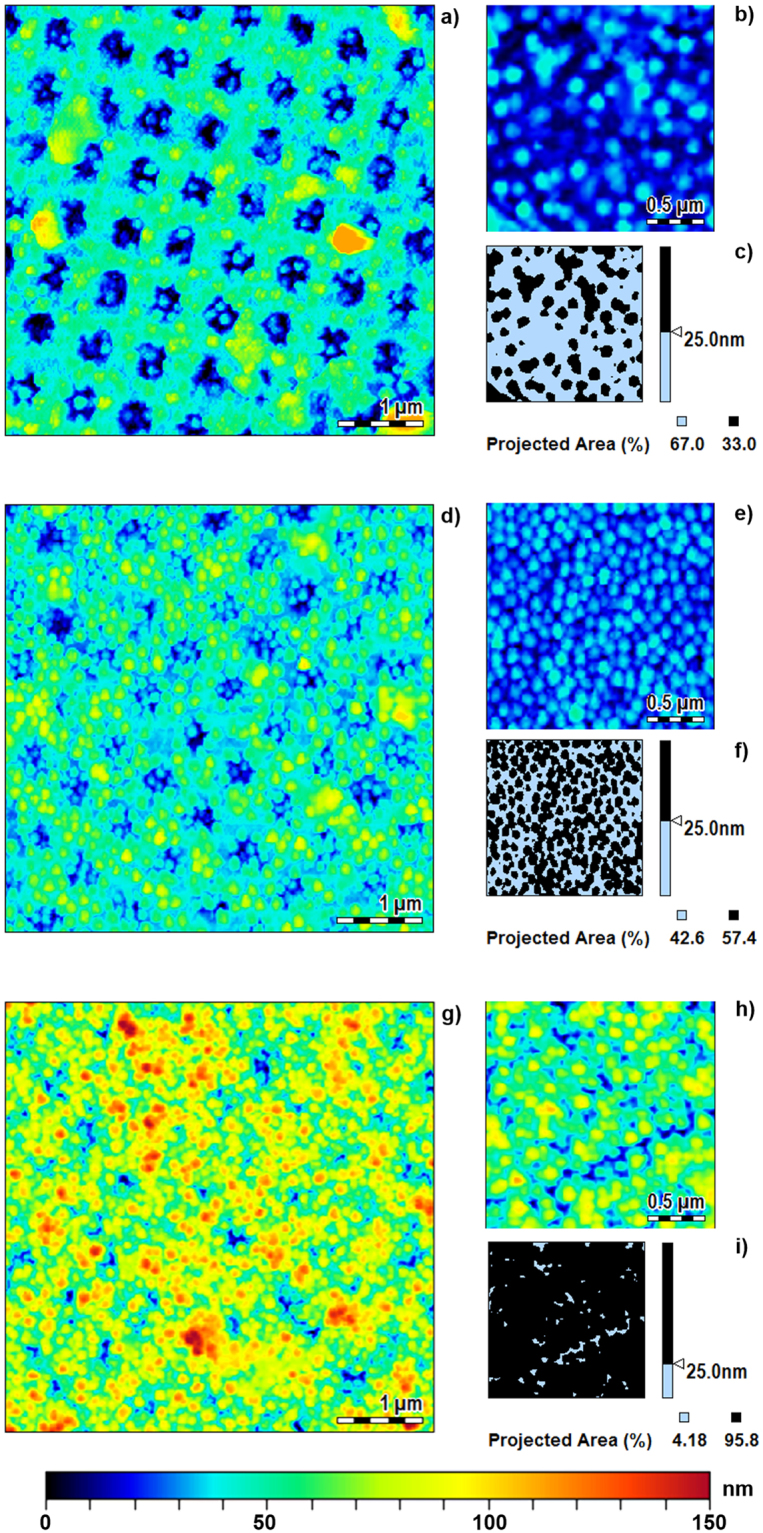
AFM characterization of the LOF probes. 5 µm × 5 µm top view around the core region of the probes integrated with MGs at concentration of 0.05% (**a**), 0.5% (**d**) and 5% (**g**). AFM images 2 µm × 2 µm top view around the unpatterned region of the probes integrated with MGs at concentration of 0.05% (**b**), 0.5% (**e**) and 5% (**h**), (**c**), (**f**), (**i**) slice processing of figures (**b**),(**e**) and (**h**) highlighting portions with thickness larger (black) and smaller(light blue) than 25 nm.

Functionalized MGs films have been morphologically characterized by means of Atomic Force Microscopy (Agilent Technologies 5420) measurements. AFM images are shown in Fig. 3(a,d,g), relative to a 5 µm × 5 µm patterned area around the fiber core. For all the concentrations, MGs look well assembled on the surface, forming a layer which follows the shape of the underlying patterned metallic slab (conformal deposition). However, for concentration of 5%, MGs look very packed among them, while at 0.05% MGs distribution is rather sparse. MGs concentration of 0.5% is a good compromise between compactness and uniformity of the resulting film. In fact, for lower MGs concentration, the MGs-Au interactions lead a strong adhesion of the particles on the free gold zones, and the MGs result sparse on the surface. By increasing MGs concentration, MGs-MGs interaction prevails and induce an increase of MGs thickness film (even creating a multilayer for higher concentrations). These differences are confirmed by the AFM analysis in a zoomed unpatterned area (2 µm × 2 µm) of the fibers tip, as shown in the Fig. 3(b,e,h). A slice processing of the previous mentioned AFM images is reported in Fig. 3(c,f,i) where a threshold of 25 nm is applied. Different MGs surface occupation of 33, 57.4 and 95.8% are found for MGs solutions of 0.05, 0.5 and 5% respectively.

**Figure 4:**
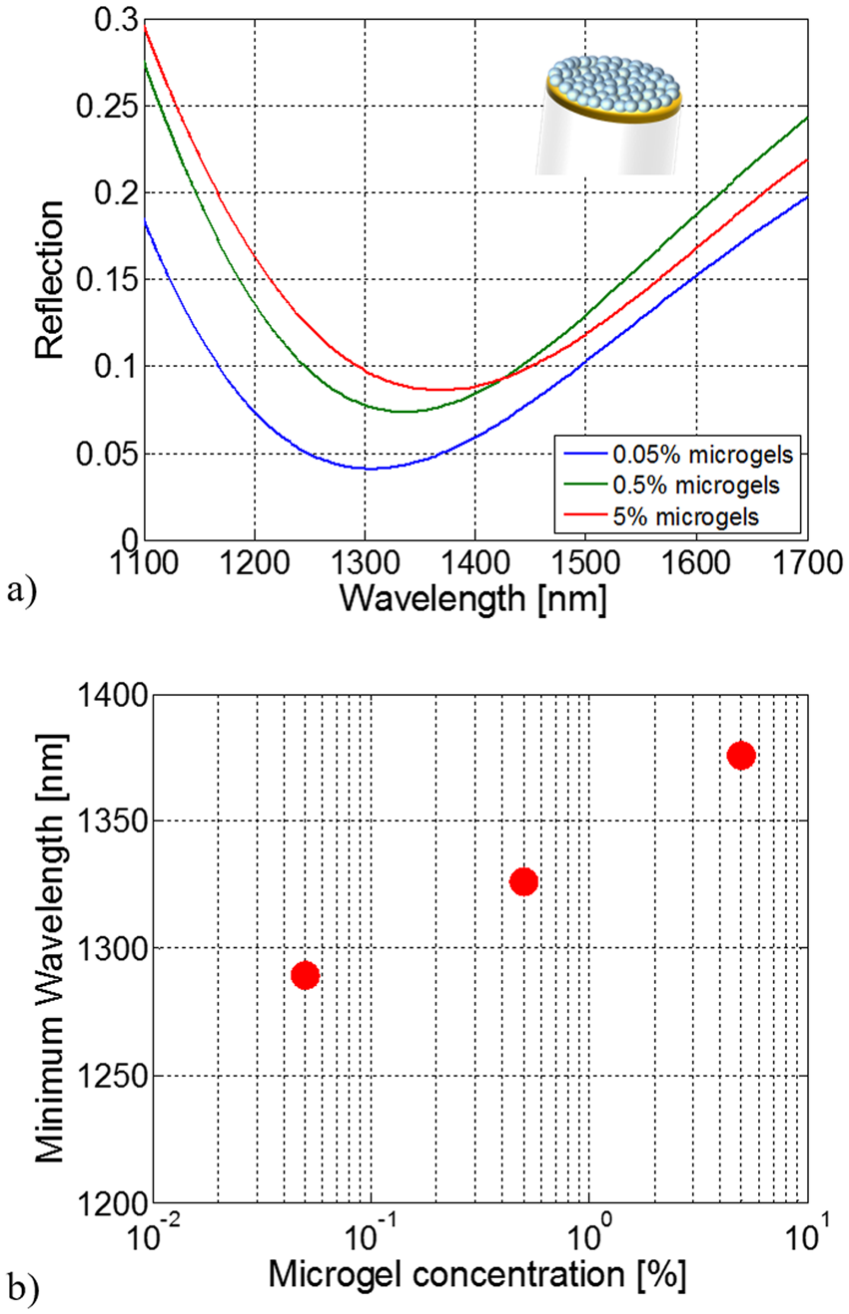
Optical characterization. Experimental reflection spectra (**a**) and their reflection dip wavelengths (**b**) of the LOF probes integrated with functionalized MGs at different concentrations.

Reflection spectra in air for the three probes are shown in Fig. 4. MGs deposition on the fiber tip induces a shift of the reflection spectrum dip of 87, 124 and 174 nm for concentrations of 0.05%, 0.5% and 5% respectively. The larger shifts achieved for higher concentrations of MGs in water solution are the result of a larger equivalent RI (increased density) or thickness (increased compactness) of the film created onto the fiber tip.

### Biological experiments: glucose detection

In order to correctly evaluate the glucose response of the integrated devices in a temperature controlled environment, the fiber probes were immersed in a cuvette (filled with 700 µL of pH 9 carbonate buffer) placed on an aluminum holder heated by a Peltier cells controlled system (Supplementary Information, section S5). In this first experiment, the buffer solution was kept at a temperature of 20 °C. Solutions at different glucose concentrations (16 µM, 160 µM, 1.6 mM and 16 mM) were directly injected in the cuvette, thus avoiding the contact of the sensible area with air. Reflection spectra were recorded every minute. For each glucose concentration, spectra acquisitions have been stopped when wavelength shift changes between two adjacent points were less than 1%.

**Figure 5:**
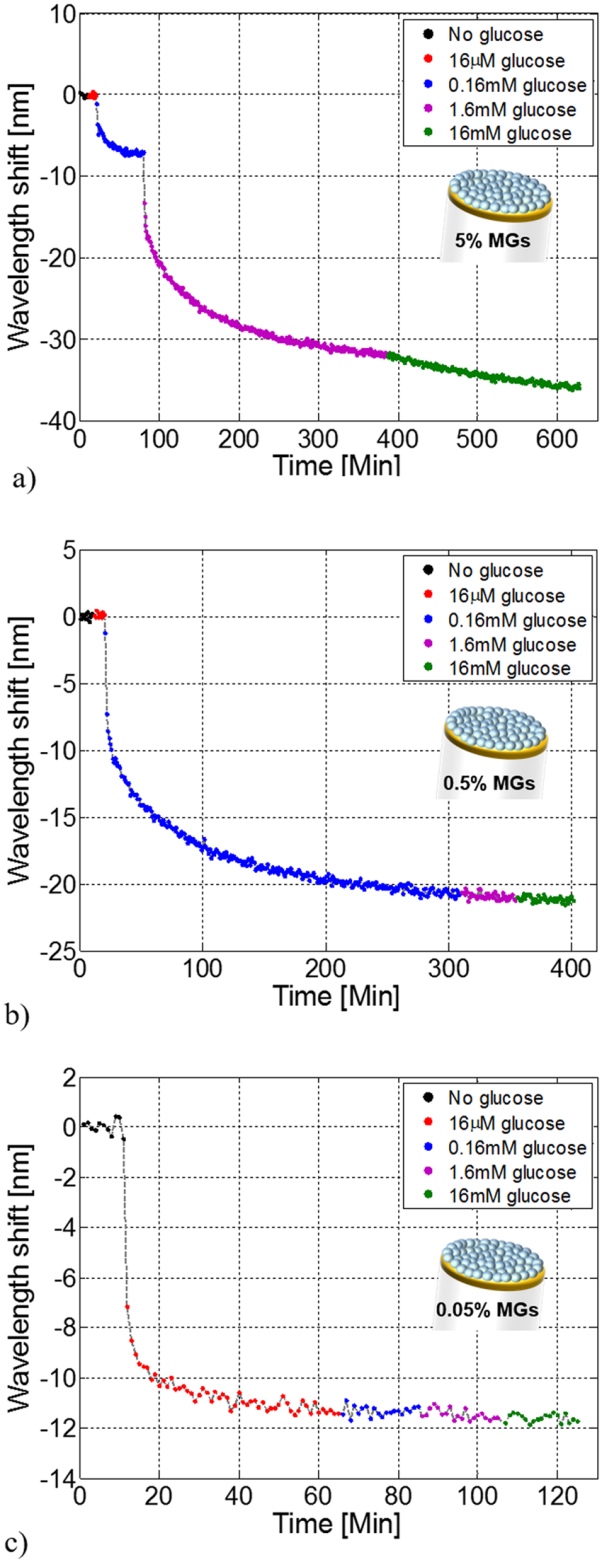
Sensorgrams of LOF devices integrated with functionalized MGs at different concentrations. Resonant wavelength shift as function of time at different glucose concentrations for devices integrated with functionalized MGs at concentrations of 5% (**a**) 0.5% (**b**) and 0.05% (**c**) in solution during the deposition step. All sensorgrams have been obtained at 20 °C.

Figure 5 shows the resulting sensorgrams *i*.*e*. a plot of the resonance wavelength shifts as function of time at different glucose concentrations. First, consistently with previously reported works^33,34,39,40^ we find that the SPR wavelength undergoes a blue shift in response to glucose binding. In fact, MGs swelling (caused by glucose binding) induces a significant decrease of the film density, essentially due to the decrease of polymer/liquid ratio in the gel network^40^; as the MGs layer size increases, its ‘equivalent’ RI value decreases, which induces the SPR wavelength blue shift. When the glucose concentration increases, more glucose molecules bind to the APBA-functional moieties, resulting in a further MGs layer swelling (and thus resonance blue shift) until a new equilibrium is reached. This process continues until saturation occurs.

Figure 5a shows that the probe functionalized with MGs at the highest concentration (5%) does not respond to glucose concentration of 16 µM, while wavelength blue-shifts of 7.2 nm and 31.7 nm are measured at concentrations of 0.16 mM and 1.6 mM respectively. When the concentration of 16 mM is injected in cuvette, the sensor responds by speeding up the saturation process; at that point, the sensorgram slightly changes its slope, and it finally reaches a total shift of −36 nm. The probe fabricated with MGs concentration of 0,5% (Fig. 5b) responds to glucose concentration of 0.16 mM with a wavelength shift of −20.8 nm, that is about three times higher than that previously obtained with 5% MGs probe. However, the next higher glucose concentration (1.6 mM) is not detected by the probe, which saturates faster than the previous case. Interestingly, glucose concentration of 16 µM is detected by the probe fabricated with the lowest MGs concentration, and thus characterized by the less dense MGs film. However, with reference to Fig. 5c, this sensor responds with a shift of −11.4 nm to that glucose concentration (16 µM) which had induced zero shift in the probes functionalized with both 5% and 0.5% of MGs.

These results demonstrate that the LOD is a function of the MGs film morphological properties. In other words, by acting on the MGs concentrations, it is possible to tune the sensitivity range of the biosensor, which is able to detect higher/lower glucose concentrations depending on whether the MGs concentration is high/low. In line with previously discussed DLS experiments (Fig. 2c), this effect is due to the fact that the lower glucose concentration, the smaller absolute number of molecules interacting with the MGs network. As a consequence, if the MGs density onto the fiber tip is high, the overall MGs film volume change may be not large enough to become readable by the probe. By reducing the MGs film density a larger percentage of MGs are involved in the binding process, thus giving rise to a more significant volume change.

Besides this analogy, a substantial difference arises between DLS and optical probe measurements. With reference to Fig. 2c, MGs at concentrations of 1 mg/mL and 0.1 mg/mL exhibit the *same* swelling at saturation (R_h_~90 nm), but for different glucose concentrations. On the other hand, optical measurements with the probes reveal that the higher is the MGs concentration, the higher is the measured wavelength shift at saturation. This phenomenon can be explained with the plasmonic nature of the LOF probe. In fact, a denser MGs film may be interpreted as layer with either larger equivalent RI (for a given thickness) or higher thickness (for a fixed RI). In the first case, swelling-induced RI variations starting from a larger initial RI induce larger SPR wavelength shifts. In the second case, the thickness increase occurring around a larger MGs layer thickness gives rise to larger resonant wavelength blue-shift.

Numerical simulations sustaining this conjecture have been included in the Supplementary Information (section S2). In this framework, the tuning of the evanescent field tail of the SPR, achieved by acting on the nanostructure integrated on the fiber tip, can be considered another important degree of freedom for controlling the detection capabilities of our probe.

The overall wavelength shifts ranging from about 10 to 40 nm measured in the detection experiments, are quite remarkable in relation to the small dimensions (180 dalton) of glucose molecules. For trying to correctly evaluate the sensitivity enhancement due to the integration of the MGs layer, we carried out glucose detection experiments by using the LOF probe without MGs integration. This test is a sort of reference for standard label free approaches. For making the comparison fair, we adopted the same functionalization procedure (based on APBA) used to functionalize MGs. The achieved sensorgram (Fig. S6.1) demonstrates that LOF device does not respond to any of the glucose concentrations considered in our analysis (see Supplementary Information, section S6). This is because the glucose molecule size is so small that the surface modifications on the fiber tip are not able to induce significant variations on the sensor surface. Moreover, for completeness, we have also experimentally evaluated the sensorgrams relative to the device integrated with functionalized MGs not activated by EDC (negative control); as expected, no wavelength shift is observed (flat sensorgrams for negative controls are shown in Fig. S6.2). These observations also represent an evidence that bulk refractive index variations of buffer solutions with different glucose concentrations do not affect the sensor response to glucose.

Another key aspect to note is the time response to glucose detection. Although providing higher absolute values of resonant wavelength shifts, the probes fabricated starting from higher MGs concentrations in water solution are characterized by longer time responses. This effect can be explained by considering that the higher is the density and compactness of the MGs film, the higher is the difficulty of the glucose molecule to permeate inside the polymeric network, and thus more time is required to reach the reaction equilibrium. Clearly, DLS measurements, being the MGs dispersed in buffer solution, are not affected by this trade-off between maximum variation and time response. Overall, response time of the order of tens of minutes represents a significant point of strength of MGs with respect to ‘bulk’ layers such as HGs, which are generally characterized by response time that are more than two orders of magnitude larger^41^.

**Figure 6:**
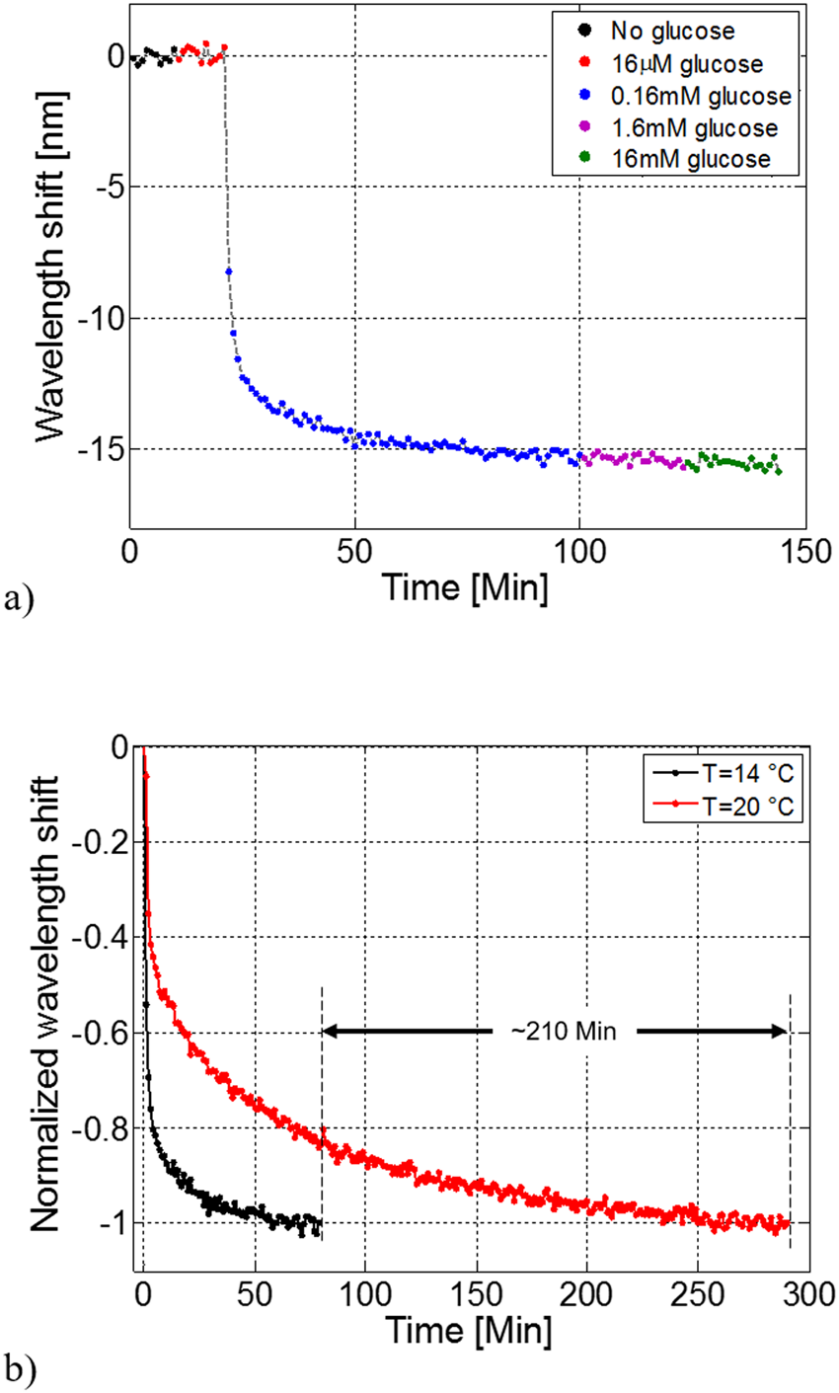
Sensorgrams of LOF device integrated with functionalized MGs at concentration of 0.5%. Resonant wavelength shift as a function of time for different glucose concentrations at 14 °C. (**a**) Comparison between sensorgrams of Fig. 5a and b for 0.16 mM glucose concentration: resonant wavelength shifts have been normalized with respect to their maximum values (**b**).

Moreover, as previously reported in Fig. 3b, response time can be also tuned by acting on the MGs temperature during the binding step. We have thus repeated the same glucose binding detection experiment at 14 °C, (i.e. 6 degrees lower than the previous one) by using the *same* probe. Results are shown in Fig. 6a. Consistently with the DLS measurements, a shorter response time is achieved at the expense of a lower sensitivity (smaller absolute wavelength shift). In particular, by comparing sensorgrams of Fig. 5a and b, it results that the probes working at 14 and 20 °C detect the same glucose concentration (0.16 mM), and then saturate after about 100 and 300 minutes respectively. However, by working at 14 °C, such a concentration induces an overall shift of 16 nm that is about 5 nm smaller than that measured at 20 °C. For a better comparison of the different response times, we report in Fig. 6b, for both the temperatures, the wavelength shifts achieved during the 0.16 mM glucose binding step normalized to their maximum, where different kinetics are evident.

## Conclusion

A biosensing platform resulting from the integration between plasmonic LOF device and functionalized MGs has been reported. With particular reference to small molecule detection, we have demonstrated that MGs deposited on top of optical fiber tip enable concentration of binding events through the use of their tridimensional network, as well as the amplification of the optical response of the probe through their swelling in response to molecule binding. Resonance shifts of the order of a few tens of nanometers can be achieved at glucose millimolar concentrations. Remarkably, such concentrations are not detected by using the same probe working with a standard label-free approach (i.e. without MGs integration). We have successfully demonstrated that, by exploiting the degrees of freedom offered by MGs, it is possible to tune the detection properties in terms of sensitivity range and response time. In particular, we have found that by choosing higher/lower concentrations of MGs in solution used during the probe fabrication procedure it is possible to detect lower/higher glucose concentrations, thus moving toward higher/lower values the LOD. Interestingly, we have also found that a trade-off exists between maximum available wavelength shift (sensitivity) and response time, with the latter that can be adjusted by changing the temperature of MGs in solution during the binding phase.

The developed platform offers many other degrees of freedom for optimizing the sensing capabilities. As an example, by changing monomer cocktail, cross linker, surfactant concentration and synthetic procedure, different kind of MGs in terms of size and thus swelling properties can be obtained^21,23,27^. For example, by having in mind *in-vivo* operation, MGs can be synthesized in order to make them completely insensitive to temperature, in such a way that the wavelength shifts measured inside the human body are only due to target molecule binding. Commonly used temperature-insensitive gel-based particles include: poly(lactic co-glycolic acid) (PLGA), poly(ethylene glycol) (PEG), poly(hydroxyethyl methacrylate) (PHEMA) and poly(vinyl alcohol) (PVA)^42,43^ or functionalized glycopolymer^44^. Concerning sensor regeneration for multiple analysis, although the gel network may affect the full reversibility, the molecule-ligand interaction is generally reversible^41^. Obviously, the regeneration procedure has to be optimized from time to time depending on the MGs typology, functionalization protocol and target molecule.

Future studies will be focused on optimizing procedures for controlling MGs deposition. Although in our experiments the EOT-like structure parameters have been chosen in order to maximize the sensitivity at the expenses of the resonance bandwidth, resonance Q factor and sensitivity can be significantly optimized through exploitation of advanced patterns and structures^6,45,46^.

Overall our results demonstrate that the combination of LOF device with MGs represents an effective technological paradigm for label-free biosensing. The improved detection performances resulting from the binding response amplification is particularly useful in the case of small molecules detection. Although here demonstrated for glucose detection, the versatility characterizing MGs make this platform easily extendable to the analyte detection cases where standard approaches fail.

## Electronic supplementary material

Supplementary Information
