# Supplementary material for: Microgel assisted Lab-on-Fiber Optrode
Source: Sci Rep. 2017 Oct 31;7:14459. doi: 10.1038/s41598-017-14852-5 (PMC5663859; doi:10.1038/s41598-017-14852-5)
Supplement: Supplementary file 1 — Supplementary Information [file 41598_2017_14852_MOESM1_ESM.pdf]

Microgel assisted Lab-on-Fiber Optrode

Supplementary Information

A. Aliberti[1], A. Ricciardi[1], M. Giaquinto[1], A. Micco[1], E. Bobeico[2], V. La Ferrara[2], M. Ruvo[3], A. Cutolo[1], A. Cusano[1,*]

[1] Optoelectronics Group, Department of Engineering, University of Sannio, I-82100, Benevento, Italy

[2] ENEA, Portici Research Center, P.le E. Fermi 1, 80055 Portici, Napoli, Italy

[3] Institute of Biostructure and Bioimaging, National Research Council, I-80143, Napoli, Italy

[*]*corresponding author: a.cusano@unisannio.it*

S1. MGs functionalization

5 mL of purified P(NIPAM-AAc) MGs was cooled at 4°C and added to a solution of 250mM of EDC and 125mM APBA in pH 4.7 MES buffered saline. The solution was cooled at 4°C, and the reaction mixture was allowed to proceed overnight at 4°C. The products were purified by dialysis against water. Negative control samples were similarly prepared by using P(NIPAM-AAc) MGs reacted with APBA only (without EDC) in pH 4.7 MES buffer.

S2. Numerical simulations

For the numerical analysis we used the commercial software COMSOL Multiphysics (RF module) based on the finite element method [1]. Following a similar approach of ref. [2], by exploiting the translational symmetries, the computational domain has been reduced to the unit cell corresponding to the lattice period, terminated with Floquet periodic boundary conditions, placed two-by-two in the opposite walls. The resulting structure supports a transverse electromagnetic (TEM) wave emulating the normally incident plane wave; we have used this kind of excitation instead of a proper fiber mode with Gaussian profile to simplify our simulations. Gold and silica have been modeled with dispersive refractive index functions taken respectively from [3] and [4]. In Fig. S2.1, the cross section view of the computational domain is shown. In the wake of our AFM morphological measurements, we have considered the patterned hole sidewalls tilted of an angle γ_1 with respect to fiber tip plane.

With reference to Fig. S2.1, the gold and chromium layers thickness are respectively h_g and h_c, while d is the hole diameter evaluated at half of total metallic layer thickness (h_g+ h_c). A conformal dielectric layer

on the metallic nanostructure has been considered in our model, with the possibility to control its thickness h_d and its hole sidewall inclination.

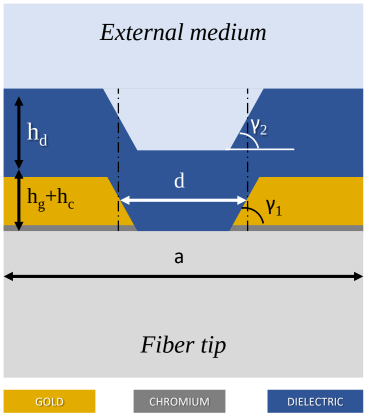

Fig. S2.1. *Cross section of the computational domain, corresponding to the grating single cell.*

By taking into account fabrication tolerances, the structure has been modeled by setting a=650nm, FF=0.3, d=2·FF·a=390nm, h_g=33nm, h_c=5nm, γ_1= γ_2=30°. Sensitivities of the standard EOT configuration (i.e. the structure without MGs deposition) have been evaluated. In that case, the dielectric layer mimics the presence of a bio-molecules on the substrate, and has been modeled considering *a* refractive index equal to 1.45. In order to evaluate the surface sensitivity we considered the resonance minimum position as function of biological layer thickness, varying in the range between 5nm and 30nm, end extracting the interpolation line slope (Fig. S2.2(a)). Bulk refractive index sensitivity has been evaluated with a similar method, but removing the dielectric layer, and changing the external medium refractive index from 1.33 to 1.34 (Fig. S2.2(b)).

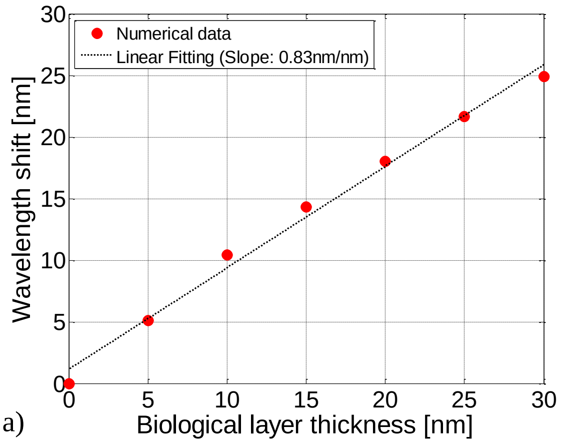

a)

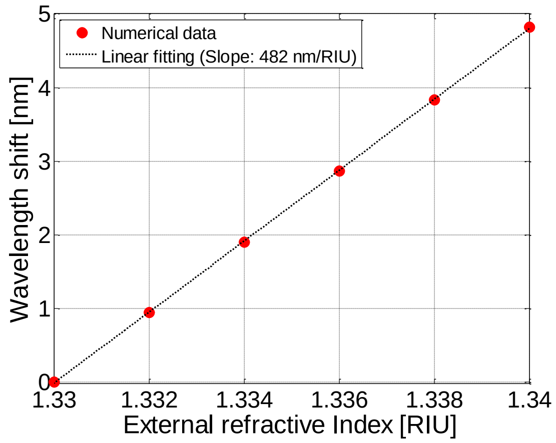

b)

Fig.S2.2. *Resonance wavelength shift numerically evaluated as function of biological layer thickness ($n_{external}$=1.33, n_{layer}=1.45) (a) and external refractive index (no layer), with a=650nm, FF=0.3, d=2·FF·a=390nm, h_g=33nm, h_c=5nm, γ_1= γ_2=30 (b).*

With the assumption that the MGs deposited on the nanostructure constitutes a uniform layer with an average refractive index, the effect of the MGs swelling followed by the consequential refractive index change have been simulated keeping fixed the structure morphological parameters.

Fig. S2.3(a) shows the resonant wavelength as function of the MGs layer thickness, in the range 0-20nm, for three different values of the refractive index. As expected, the sensitivity to dielectric thickness changes (curves slope) increases by increasing the slab refractive index, and saturates starting from a thickness of about 150nm, dependently on the plasmonic mode field distribution. The overall resonant wavelength shift could be described by a path joining the points thickness/RI on each curve, by taking in consideration that the refractive index decreases with the thickness increase. Fig. S2.3(b) shows the dual results, i.e. the resonant wavelength as a function of the MGs

layer refractive index, in the range 1.35-1.45 [5 - 6], for three different values of the slab thickness. Accordingly to Fig. S2.3(a), an increase of the MGs slab thickness determines a higher value of sensitivity to refractive index changes.

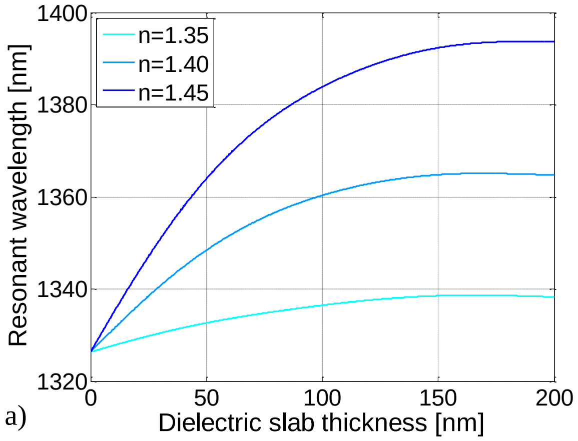

a)

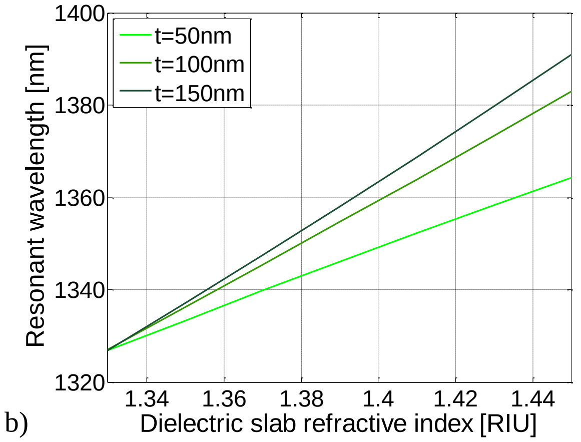

b)

Fig. S2.3. *Resonant wavelength as function of MGs layer thickness for three different values of the refractive index (a) and as a function of the MGs layer refractive index for three different values of the slab thickness (b) with a=650nm, FF=0.3, d=2·FF·a=390nm,* h_g*=33nm,* h_c*=5nm,* $\gamma_1 = \gamma_2$*=30,* $n_{external}$*=1.33.*

S3. Optical characterizations

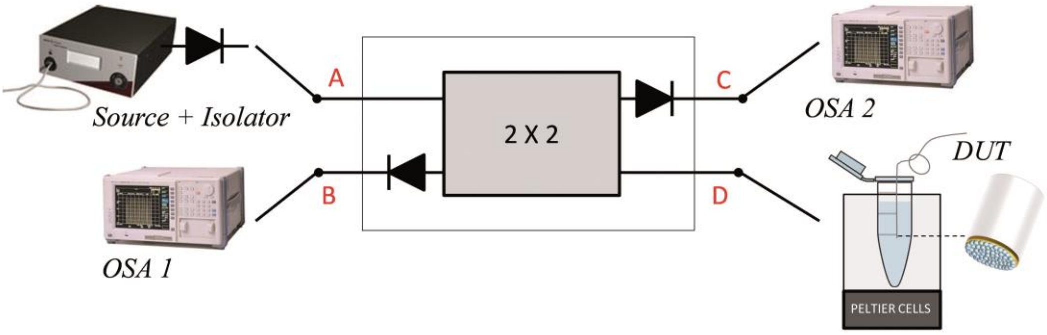

Fig. S3.1.*Schematic of the experimental setup used for optical characterizations.*

Fig. S3.1 shows a schematic of the experimental setup used for optical characterizations. The setup is composed of a broadband optical source (NKT SuperK COMPACT), a 2×2 coupler, two Optical Spectrum Analyzer (OSA1: Ando AQ6315B, OSA2: Ando AQ6317C), three isolators, and an Eppendorf tube holder heated by Peltier cells control system. Specifically the source is a supercontinuum lasers with its collimator inserted into a splitter that provides two outputs with different wavelength ranges. The IR output (800-2400 nm) is connected to a fiber collimator through a coupler equipped by alignment mirrors. With reference to Fig. S3.1, a first isolator is placed in output to source, while the other two are used just before the two OSA in order to avoid spurious reflected components coupled in the optical circuit. The reflected spectrum is given by the ratio between the data acquired from OSA1 and OSA2 respectively, normalized with respect to the transfer functions of the paths AC, and ADB of the coupler. The normalization allows to have a spectrum not influenced by the paths asymmetry and by both the coupler and isolators dispersive behavior. A Labview plug-in as been used for the OSA scan timing control (via GPIB interface protocol), ensuring the acquisitions simultaneity, and consequently improving the robustness against the source undesired oscillations.

Fig. S3.2 shows the measured reflection spectrum of the fabricated LOF probe and a comparison with respect to the numerical counterpart. A SEM image (top view) is also shown.

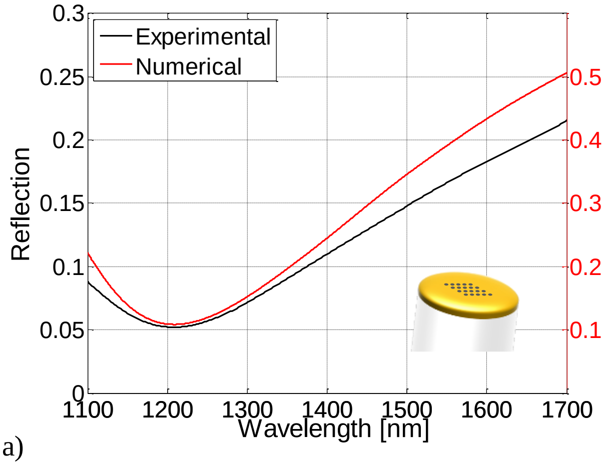

a)

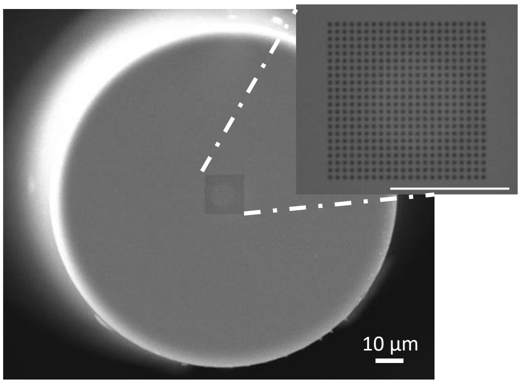

b)

Fig. S3.2. *LOF probe characterization. Numerical (red) and experimental (black) reflection spectrum of the LOF probe before MGs deposition (a), SEM image (top view) of the fabricated probe before MGs deposition (b).*

S4. DLS measurements

Measurements were conducted using a DLS system (Malvern Zetasizer Nano ZS instrument, 633 nm laser, 173° scattering angle) equipped with a temperature controller. For thermo-responsivity measurements, an equilibration time of 1200s was used for each temperature and a total of 5 run were conducted. The (pNIPAm-co-AAc) MGs measurements were performed in water at pH 4 adjusted by using dilute HCl solution. The (pNIPAm-APBA) MGs and (pNIPAm-APBA+Glucose 16mM) MGs were performed at pH 9 carbonate buffer because at lower pH the majority of PBA groups are in the uncharged form, and as a result, they lose their glucose responsibility under this conditions.

For glucose binding assay at 14°c and 20°C,after an equilibration time of 1200s, 10µL of glucose solution (1.60M) was quickly injected into a 990µL of MGs dispersion (1mg/mL) in sodium carbonate buffer and data acquired with a time step of 180 seconds. For binding assay with different MGs concentration (1mg/mL and 0.1mg/mL) at 20°C, after an equilibration time of 1200s, 2 aliquots of 10µL of glucose solution were added in order to reach a final glucose concentrations of 16mM and 1.6mM and data acquired with a time step of 180 seconds.

S5. Description of the chamber for thermal measurements

In the Fig. S5 is shown the Eppendorf tube holder enabling the temperature control of the experimental environment.

It is composed of a brass die machined with a CNC mill that match perfectly the Eppendorf tube shape. The die is in contact with six Peltier cells (three for each side) that cool/heat the solution inside the tube. Two heat sinks are placed on the opposite face of the Peltier cells in order to dissipate the heat produced by the cells under the cooling phase, and thus keeping high the efficiency of the cells. A Teflon support is machined in order to align the Peltier cells on the die and on the heatsink and support the die in the center, and for the thermal insulation of the die respect the external environment and heatsinks. A range between 0°C and 55°C can be set inside the Eppendorf tube at room temperatures in the surrounding environment.

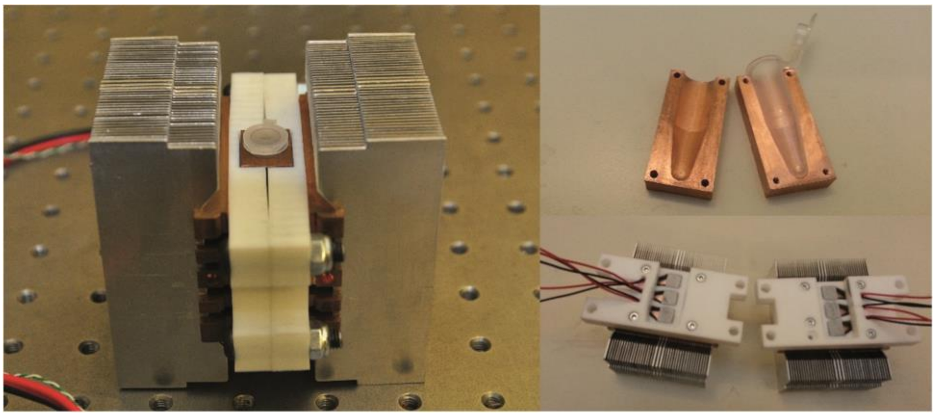

Fig. S5. *Eppendorf tube holder heated by Peltier cells control system.*

S6. Negative controls: Glucose detection with LOF probe without MG and with not-activated MGs.

We carried out glucose detection experiments by using the LOF probe without MGs integration. This test is a sort of reference and it is useful for trying to correctly evaluate the sensitivity enhancement due to the integration of the MGs layer. To this aim, in order to make the LOF device receptive to glucose molecules, we adopted the same functionalization procedure used to functionalize MGs based on APBA. Specifically, the probe was immersed into an ethanol solution of 10 mM of 11-mercaptoundecanoic acid for 6 h and dried under a nitrogen atmosphere. The probe was then placed in pH 4.7 MES buffered for 1 h at 4°C into the refrigerator and a solution of 250mM EDC and 125mM APBA was added to the buffer. The reaction proceeded overnight at 4 °C.

During biological assay, APBA functionalized LOF probe was immersed in a cuvette filled with pH 9 carbonate buffer solution. Solutions at different glucose concentrations (16μM, 160 μM, 1,6mM and 16mM) were directly injected in the cuvette, thus avoiding the contact of the sensible area with air. In other words, the fiber probe was always immersed in liquid solutions during the whole experiment. Reflection spectra were recorded every minute. The results are summarized in Fig. S6.1, where the resonance wavelength shift as a function of time are shown. It results clear that the LOF device does not exhibit any response to all glucose concentrations considered in our analysis.

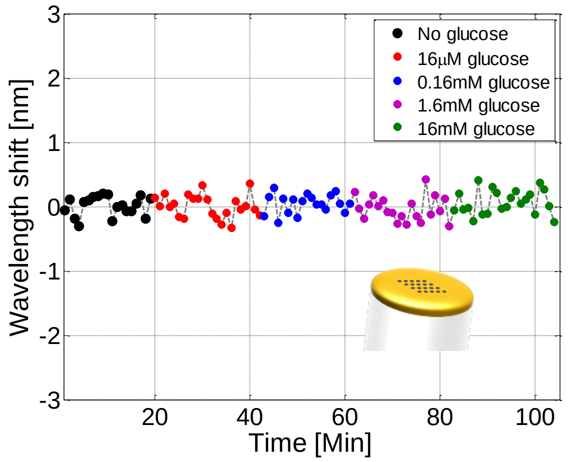

Fig.S6.1: *Resonant wavelength shift as function of time at different glucose concentrations for LOF probe without MGs*

It is important to remark that the effectiveness of the functionalization protocol, and the consequent glucose binding on the gold surface, have been confirmed by an enzymatic colorimetric assay

carried out on the fiber probe (see next section S7 of the supplementary information for more details).

We also experimentally evaluated the sensorgrams relative to the device integrated with functionalized MGs but not activated by EDC (negative control); Also in this case, as shown in figure S6.2, without the boronic acid acting as ligand, no wavelength shift is observed.

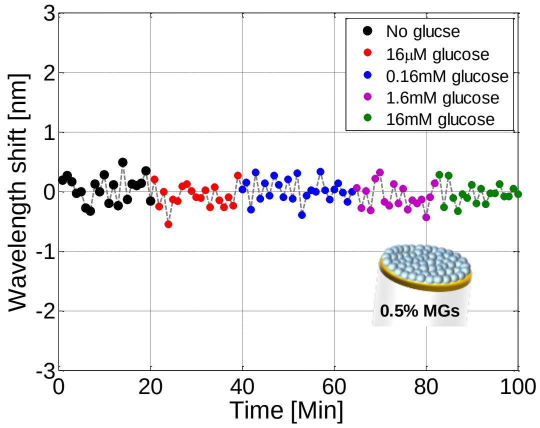

Fig. S6.2*: Wavelength shift as function of time at different glucose concentrations for LOF probe with not activated MGs at 0.5% (negative control).*

S7 Glucose assay kit protocol (enzymatic colorimetric assay)

The coupling of APBA on gold surface and its capability to bind glucose molecules was evaluated by using a Glucose (GO) Assay Kit purchased from Sigma Aldrich. The colorimetric assay involved the following steps:

i) glucose is oxidized to gluconic acid and hydrogen peroxide by glucose oxidase.;

ii) hydrogen peroxide reacts with o-dianisidine in the presence of peroxidase to form a colored product;

iii) oxidized o-dianisidine reacts with sulfuric acid to form a stable colored product (pink color). The intensity of the pink color is proportional to the original glucose concentration.

To perform the colorimetric assay, the optical fiber probes was dipped in a standard glucose solution [1mg/ml] for 30 minutes. The probes were then placed in a tube containing 5μL of carbonate buffer and 10μL of Assay Reagent for 30 minutes at 37 °C. The reaction was stopped by adding 10μL of 12N H_2SO_4 into each tube.

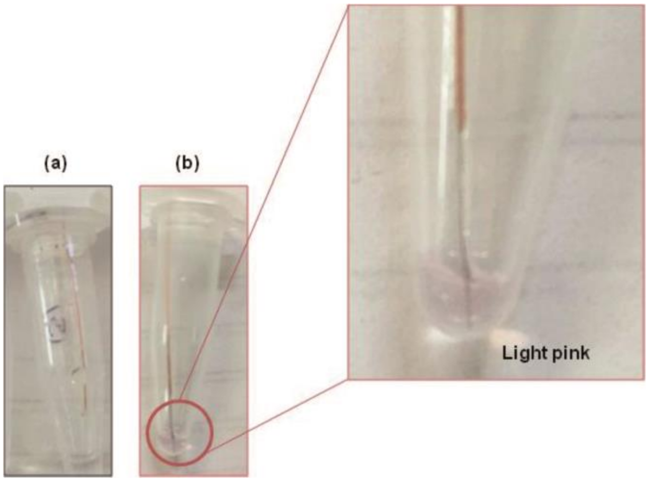

Fig.S7*: Photographs of the assay reaction tubes for negative control (a) and APBA functionalised probe (b).*

The probe functionalized with APBA showed a light pink color that confirmed the presence of glucose molecules bound to the boronic acid moieties. For the negative control, the pink color production was not observed: in absence of APBA glucose molecules are not able to bind to gold surface.

References

1. ComsolMultiphysics. In *RF User's Guide, version 3.5a*. Comsol AB: Stockolm, (2008).
2. Consales, M.,Ricciardi, A.,Crescitelli, A., Esposito, E.,Cutolo, A.,Cusano A. Lab-On-Fiber Technology: Toward Multifunctional Optical Nanoprobes. *ACS nano*, **6**, 3163-3170(2012).
3. Palik, E. D. *Handbook of Optical Constants of Solids*. Academic Press: Orlando (1985).
4. Malitson, H. Interspecimen Comparison of the Refractive Index of Fused Silica, *J. Opt. Soc. Am.***55**, 1205-1208(1965).
5. Sorrell, C. D.,Serpe, M. J. Reflection Order Selectivity of Color-Tunable Poly (N-Isopropylacrylamide) Microgel Based Etalons. *Adv. Mater.***23**, 4088-4092 (2011).
6. Reufer, M., Diaz-Leyva, P., Lynch, I.,Scheffold, F. Temperature-Sensitive Poly (N-Isopropyl-Acrylamide) Microgel Particles: A Light Scattering Study. *Eur. Phys. J. E: Soft Matter Biol. Phys.***28**, 165-171 (2009).
